# Association between lifestyle and thyroid dysfunction: a cross-sectional epidemiologic study in the She ethnic minority group of Fujian Province in China

**DOI:** 10.1186/s12902-019-0414-z

**Published:** 2019-07-30

**Authors:** Yanling Huang, Liangchun Cai, Yuanyuan Zheng, Jinxing Pan, Liantao Li, Liyao Zong, Wei Lin, Jixing Liang, Huibin Huang, Junping Wen, Gang Chen

**Affiliations:** 1 Department of Endocrinology and Metabolism, Zhongshan Hospital Xiamen University, 201-209 Hubin South Road, Xiamen, 361004 China; 2 Department of Endocrinology, Shengli Clinical Medical College of Fujian Medical University, Fuzhou, 350001 China; 3 Department of Geriatrics, Shengli Clinical Medical College of Fujian Medical University, Fuzhou, 350001 China; 4 Department of Endocrinology, Fujian Provincial Hospital, Fujian Academy of Medical Sciences, Fuzhou, 350001 China

**Keywords:** Life style, Epidemiologic study, Hyperthyroidism, Hypothyroidism, Thyroid peroxidase antibody (TPOAb)

## Abstract

**Background:**

Thyroid dysfunction is one of the prevalent endocrine disorders. The relationship between lifestyle factors and thyroid dysfunction was not clear and some of the factors seemed paradoxical.

**Methods:**

We conducted this population-based study using data from 5154 *She* ethnic minority people who had entered into the epidemic survey of diabetes between July 2007 to September 2009. Life style information was collected using a standard questionnaire. Body mass index (BMI), Blood pressure and serum TSH, TPOAb, triglycerides (TG), total cholesterol (TC) and high–density lipoprotein cholesterol (HDL) were collected.

**Results:**

The study showed that people who drank, had higher education or suffered from insomnia have lower incidence of hyperthyroidism. On the other hand, smoking, alcohol consumption, exercise, undergoing weight watch and chronic headache were associated with decreased incidence of hypothyroidism. Using multivariable logistic regression analysis, we found that alcohol consumption was associated with decreased probability of hyperthyroidism, hypothyroidism, as well as positive TPOAb. The amounts of cigarettes smoked daily displayed a positive correlation with hyperthyroidism among smokers. Accordingly, smoking seemed to be associated with decreased risk for hypothyroidism and positive TPOAb. Exercise and maintaining a healthy weight might have a beneficial effect on thyroid health. Interestingly, daily staple amount showed an inverse correlation with incidence of positive TPOAb.

**Conclusions:**

Within the Chinese *She* ethnic minority, we found associations between different lifestyle factors and the incidence of different thyroid diseases. Understanding the nature of these associations requires further investigations.

## Background

Thyroid dysfunction is one of the prevalent endocrine disorders. Thyroid dysfunction can lead to various health issues including cardiovascular disorders [1], abnormal glucose intolerance, impairment of liver function, dyslipidemia, and so on. Thyroid dysfunction may also cause psychological symptoms and affect quality of life [2]. It was found that even at subclinical level, hyperthyroidism and hypothyroidism are associated with overall reduction of medical well-being [3].

The observation of different prevalence rate among different ethnic groups and geological areas indicates that both genetic and environmental factors contribute to the development of thyroid dysfunction. It is well known that most of the thyroid dysfunction is caused by autoimmune thyroid diseases (AITD). Thus it is not surprising that polymorphisms in major histocompatibility genes (human leukocyte antigen [*HLA*]), immunoregulatory genes (*CTLA4, PTPN22, FOXP3, CD25, CD40, FRCL3*), and thyroid specific genes (*TSHR*,*TG*) were found to be associated with increased prevalence of the disease [4]. On the other hand, There are also indications that environmental factors such as alcohol consumption, smoking, Iodine intake, deficiency in vitamin and minerals such as Vitamin D and Selenium, infections, stress, and certain drugs (estrogens) may also affect the incidence of the thyroid dysfunction [5]. How different environmental and lifestyle factors affect development of thyroid disease is not well understood. Interestingly, recent studies have indicated that environmental and lifestyle factors may interact with genetic factors by influencing epigenetic mechanisms, such as altering DNA methylation and chromatin modifications, thereby modulating gene expression [6]. Studies from many areas around the world have investigated potential environmental risk factors of thyroid dysfunction. However, some of the results remain controversial. For example, a survey by Nystrom et al. (1993) identified smoking as a risk factor for hypothyroidism [7], while other studies failed to find any association [8]. A recent research in fact found that smoking might reduce the risk of hypothyroidism [9]. Besides smoking, other lifestyle factors need to be explored to identify additional contributors to the disease development.

We have the complete dataset of an epidemic survey of diabetes in the *She* ethnic minority group of Fujian Province in China, which was carried out from July 2007 to September 2009. The details of this study have been published previously [10–12]. Among the participants, 5154 were tested for the TSH and TPOAb levels. Using these data, we conducted statistical analyses to investigate potential associations between several lifestyle factors and the incidence of thyroid dysfunction.

## Methods

### Study design and population

The *She* ethnic minority is one of the smaller ethnic populations in China. Confining our study to such a cohort with relatively homogenous genetic and general environmental background allows us to better isolate lifestyle factors from the genetic components of the disease, as well as minimize the effect of other hidden environmental variables. The study is part of an epidemic survey of the diabetes in the *She* ethnic minority group of Fujian Province. We recruited participants by advertising and phone calls made by the community workers. Subjects registered were permanent residents of the Fujian province *She* reservation area, aged 20–80 years. A multistage, stratified, cluster random sampling method was used in order to select a representative sample. A total of 5385 *She* ethnic residents were enrolled in the survey and 5154 subjects who had results of TSH and TPOAb levels were collected in our study. The study was approved by the Ethics Committee of Fujian Medical University. We had complied with the Declaration of Helsinki Ethical Principles for medical research involving human subjects [13]. All the participants signed informed consent authorized by the Chinese Medical Association before data collection.

### Patient and public involvement

The role of subjects in this study were participants. They were not involved in the recruitment or conduction of the study. After the completion of the study, we sent each participant a letter describing the detailed results of their own physical examinations and lab tests free of charge. If the lab tests or physical examinations showed abnormal results, we informed the participants by phone calls and advised them to go to the clinic for appropriate further examination and treatments.

### Data collection

Subjects were individually interviewed by trained staff. During the interview, life style information was collected using a standard questionnaire. Smokers were defined as those who were having or had had at least 5 cigarettes per day. The numbers of cigarettes taken by the smokers were also recorded. As the *She* minority group use cereal as their staple food, daily staple was defined as the amount of cereal consumed per day. Alcohol consumption was defined as the average consumption of at least 35 g of alcohol per day. Salt intake was an estimation of the individual’s salt intake per year. Due to mandatory iodization of salt in the area, salt intake was a proxy for iodine intake. Exercise was self-reported, defined as performing physical activities for at least 20–30 min twice or more per week. Dietary restriction was defined as being on a low fat and low-calorie diet. Frequent sugary beverages referred to consuming sugary beverages more than twice a week. People who measured their body weight more than once a week were characterized as “yes” for weight watching. Chronic insomnia referred to having disrupted sleep that occurred at least three nights per week and lasting for at least three months. Chronic daily headache was defined as having a headache for more than four hours on more than 15 days per month. In addition to the above life style information, the accompany diseases and medication, family history of thyroid disease were also recorded. The participants who had already had thyroid disease were excluded.

### Physical examinations

Body mass index (BMI) was calculated as weight in kilograms divided by height in meters squared (kg/m^2^). Blood pressure was measured twice in the sitting position by manual sphygmomanometer on the right arm after at least 5 min of rest, and the mean of the two readings was used for analysis. All of the staffs conducting the physical examinations completed a training program of the entire study protocol before they began to collect data.

### Laboratory measurements

Serum TSH was assessed using an electrochemiluminescence (Nichols Institute Diagnostics, San Juan Capistrano, CA, USA). The working range for this method was 0.01–100 mIU/L. Serum TPOAb were measured with a highly sensitive, direct RIA system (Kronus, San Clemente, CA, USA). The reference range of TPOAb was ≤35 IU/ml. Serum triglycerides (TG). Total cholesterol (TC) and high–density lipoprotein cholesterol (HDL) were determined by an automatic colorimetric method (Hitachi; Boehringer Mannheim). LDL-cholesterol (LDL-c) was calculated using the Friedewald formula. All blood samples were obtained in a fasting state at 8 am.

### Diagnostic categories

Globally, the normal range of TSH level is not definitely set. Different regions, ages and ethnic groups may have different ranges. To set a reference range for normal TSH values, we selected 250 healthy participants aged 20–80 and carefully excluded those who self-reported thyroid disease or goiter, as well as those who had thyroid disease family history. We tested their TSH levels and set the range of 2.5th- 97.5th percentile as the reference range of normal TSH values. The resulting range was 0.34 mIU/L- 4.22 mIU/L. TSH level lower than 0.34 mIU/L was defined as hyperthyroidism (including subclinical hyperthyroidism). TSH level higher than 4.22 mIU/L was defined as hypothyroidism (including subclinical hypothyroidism).

### Statistical analysis

Statistical analyses were conducted using the SPSS software (version 13.0, SPSS, Chicago). The distributions of numerical variables were tested for normality. Data with normal distribution were expressed as means ± standard deviation (SD). Data with skewed distribution were log-transformed for analysis. If still with skewed distribution, data were expressed as median (interquartile range). For categorical variables, data were expressed as percentages. Student’s t-test was used for continuous variables and chi-square test for categorical variables to assess differences. Multiple logistic regression analyses were performed to evaluate factors associated with thyroid dysfunction. We set whether or not having thyroid dysfunction as the dependent variable (yes = 1, no = 0). The significant variables identified by previous Student’s t-test or chi-square test were selected to be included in the regression analyses. Continuous variables such as BMI were classified by the value of clinical significance (eg. BMI < 18.5: underweight; 18.5~23.9:normal; 24~27.9:overweight; ≥28:obesity). After testing for all possible interactions among independent variables to eliminate the influence of confounders, the best fitted logistic regression model was established to determine the possible risk factors for thyroid dysfunction. Adjusted ORs and 95% confidence intervals were calculated for all significant variables. *P* < 0.05 was considered to be statistically significant.

## Results

Out of a total of 5700 subjects initially surveyed by the questionnaire, we were able to obtain completed questionnaires with corresponding laboratory data for 5154 of them. The response rate was 90.4%. The prevalence of thyroid dysfunction was shown in the Table 1.

**Table 1:** Prevalence of thyroid dysfunction in the study

	Subjects	Abnormal TSH n (%)	Hyperthyroidism n (%)	Hypothyroidism n (%)	TPOAb positive n (%)
Male					
20–44	941	35 (3.7%)	16 (1.7%)	19 (2.0%)	48 (5.1%)
45–59	829	39 (4.7%)	23 (2.8%)	16 (1.9%)	62 (7.5%)
60–80	452	39 (8.6%)	16 (3.5%)	23((5.1%)	48 (10.6%)
All males	2222	113 (5.1%)	55 (2.5%)	58 (2.6%)	158 (7.1%)
Female					
20–44	1339	103 (7.7%)	36 (2.7%)	67 (5.0%)	178 (13.3%)
45–59	1217	93 (7.6%)	38 (3.1%)	55 (4.5%)	161 (13.2%)
60–80	376	37 (9.8%)	11 (2.9%)	26 (6.9%)	52 (13.8%)
All females	2932	233 (7.9%)	85 (2.9%)	148 (5.0%)	391 (13.3%)
All subjects	5154	346 (6.7%)	140 (2.7%)	206 (4.0%)	549 (10.7%)

Among these 5154 participants, based on TSH level, 140 (2.72%) were diagnosed with hyperthyroidism (aged from 20 to 80) and 206 (4.00%) were diagnosed with hypothyroidism (aged from 20 to 80). Incidence of TPOAb positive was 10.65%. The characteristics of the subjects categorized by thyroid status were presented in Table 2.

**Table 2:** Characteristics of the subjects categorized by thyroid status

	Euthyroidism (*n* = 4798)	Hyperthyroidism (*n* = 140)	Hypothyroidism (*n* = 206)	TPOAb negative (*n* = 4605)	TPOAb positive (*n* = 649)
Age (years)	46.62 ± 12.64	49.17 ± 11.43^a^	48.00 ± 14.42	46.62 ± 12.73	47.88 ± 12.23^d^
Sex (male/female)	2109/2699	55/85	58/148^c^	2064/2541	158/391^f^
BMI (Kg/m^2^)	22.76 ± 3.11	22.32 ± 3.15	23.37 ± 3.79^a^	22.74 ± 3.14	23.04 ± 3.18^d^
SBP (mmHg)	134.19 ± 23.29	137.33 ± 25.08	136.67 ± 27.27	134.27 ± 23.42	135.49 ± 24.02
DBP (mmHg)	79.76 ± 13.55	80.77 ± 13.54	81.21 ± 14.26	79.80 ± 13.45	80.20 ± 12.93
HR (bpm)	74.33 ± 14.20	84.43 ± 19.24^c^	73.24 ± 13.33	74.27 ± 14.14	76.76 ± 16.11^e^
TG (mmol/L)	1.07 (0.77–1.58)	0.98 (0.70–1.42) ^a^	1.12 (0.76–1.68)	1.07 (0.77–1.59)	1.05 (0.75–1.49)
TC (mmol/L)	4.95 ± 1.38	4.69 ± 1.16^*^	5.08 ± 1.17	4.94 ± 1.39	4.99 ± 1.08
HDL (mmol/L)	1.48 ± 0.33	1.45 ± 0.36	1.51 ± 0.32	1.47 ± 0.33	1.50 ± 0.34^d^
LDL-c (mmol/L)	3.18 ± 0.87	3.00 ± 0.89^a^	3.28 ± 0.98	3.17 ± 0.87	3.23 ± 0.89
UA (umol/L)	280.58 ± 81.57	264.62 ± 80.72^a^	272.76 ± 81.75	281.46 ± 80.90	263.65 ± 75.43^f^
TSH (mIU/L)	1.44 (1.00–2.05)	0.02 (0.01–0.24) ^c^	5.69 (4.67–10.59) ^c^	1.43 (0.99–2.07)	1.80 (0.97–2.88)
TPOAb positive (%)	8.0	48.6^c^	36.4^c^		

*BMI* body mass index, *SBP* systolic blood pressure, *DBP* diastolic blood pressure, *HR* heart rate, *TG* triglycerides, *TC* total cholesterol, *HDL* high-density lipoprotein-cholesterol, *LDL-c* low-density lipoprotein-cholesterol, *UA* uric acid, Data shown as mean ± SD or %. ^a^*P* < 0.05 when comparing with euthyroidism; ^b^*P* < 0.01 when comparing with euthyroidism; ^c^*P* < 0.001 when comparing with euthyroidism; ^d^*P* < 0.05 when comparing with TPOAb negative; ^e^*P* < 0.01 when comparing with TPOAb negative; ^f^*P* < 0.001 when comparing with TPOAb negative; TG and TSH are shown as median and interquartile range

As expected, compared with people with euthyroidism, those with hyperthyroidism had higher heart rates and lower TC and LDL level. People with hypothyroidism had higher BMI. The influence of thyroid disorders on UA level was currently unclear. In our study, people with hyperthyroidism and positive TPOAb both had lower UA level. Compared with TPOAb negative, people who were TPOAb positive had higher age, BMI, HR, and HDL level.

Table 3 showed life style factors and their associations with thyroid status. As shown in the table, we found hyperthyroidism occurring less frequently among people who drank, had higher education or suffered from insomnia (*P* < 0.05 by chi square test). Incidence of hypothyroidism was decreased among people who smoked, drank alcohol, exercised, underwent weight watch, or suffered from chronic daily headache (*P* < 0.05 by chi square test). People who have higher education, smoke, consume alcohol, or undergo weight watch seem less likely to be TPOAb positive (*P* < 0.05 by chi square test). We also analyzed daily cigarette intake and daily staple food consumption among normal individuals and individuals with thyroid dysfunction. As shown in Table 3, we found that compared to people with euthyroidism, people with hyperthyroidism smoke significantly more cigarettes daily (*P* < 0.01 by student’s t-test). In addition, we found people with positive TPOAb were those who consume less daily staple food.

**Table 3:** Lifestyle factors and their associations with thyroid status

		Hyperthyroidism status			Hypothyroidism status			TPOAb status		
		Euthyroidism (%)	Hyperthyroidism (%)	*P* value	Euthyroidism (%)	Hypothyroidism (%)	*P* value	TPOAb negative (%)	TPOAb positive (%)	*P* value
		(n = 4798)	(n = 140)		(n = 4798)	(n = 206)		(n = 4605)	(n = 649)	
Daily cigarettes (number)		16.76 ± 9.63	21.38 ± 15.24	*P* = 0.003	16.76 ± 9.63	14.64 ± 6.27	*P* = 0.208	16.87 ± 9.72	16.46 ± 10.81	*P* = 0.673
Daily staple	(gram)	345 ± 138	343 ± 164	*P* = 0.862	345 ± 138	330 ± 168	*P* = 0.130	346 ± 138	332 ± 153	*P* = 0.038
Salt intake level	< 6 g/d	842 (98.1)	16 (1.9)	*P* = 0.094	842 (96.1)	34 (3.9)	*P* = 0.887	796 (89.9)	89 (10.1)	*P* = 0.621
	~ 8 g/d	1281 (97.2)	37 (2.8)		1281 (95.7)	57 (4.3)		1224 (89.5)	144 (10.5)	
	~ 10 g/d	2216 (96.7)	76 (3.3)		2216 (96.0)	92 (4.0)		2107 (88.8)	265 (11.2)	
High school education	no	834 (96.9)	15 (3.1)	*P* = 0.035	834 (96.1)	34 (3.9)	*P* = 0.774	3360 (88.7)	468 (11.3)	*P* = 0.011
	yes	3853 (98.2)	123 (1.8)		3853 (95.9)	166 (4.1)		797 (91.6)	73 (4)	
Smoker	no	3362 (97.3)	95 (2.7)	*P* = 0.555	3362 (95.1)	172 (4.9)	*P < 0.001*	3183 (88.0)	433 (12.0)	*P < 0.001*
	yes	1396 (96.9)	44 (3.1)		1396 (97.6)	34 (2.4)		1347 (92.4)	111 (7.6)	
Alcohol consumption	no	2925 (96.6)	103 (3.4)	*P* = 0.002	2925 (95.1)	150 (4.9)	*P* < 0.001	2777 (87.8)	387 (12.2)	*P < 0.001*
	yes	1788 (98.1)	34 (1.9)		1788 (97.2)	52 (2.8)		1705 (91.6)	156 (8.4)	
Exercise	no	934 (97.5)	24 (2.5)	*P* = 0.377	934 (94.5)	54 (5.5)	*P* = 0.018	896 (89.3)	107 (10.7)	*P* = 0.755
	yes	3468 (97.0)	109 (3.0)		3468 (96.2)	136 (3.8)		3288 (89.0)	407 (11.0)	
On diet	no	4436 (97.1)	131 (2.9)	*P* = 0.929	4436 (96.0)	186 (4.0)	*P* = 0.728	4226 (89.4)	527 (10.6)	*P* = 0.025
	yes	280 (97.2)	8 (2.8)		280 (95.6)	13 (4.4)		254 (85.2)	44 (14.9)	
Sugary beverages	frequently	281 (98.9)	3 (1.1)	*P* = 0.06	281 (95.6)	13 (4.4)	*P* = 0.707	258 (87.8)	36 (12.2)	*P* = 0.46
	seldom	4272 (97.0)	131 (3.0)		4272 (96.0)	177 (4.0)		4064 (89.1)	495 (10.9)	
Weight watch	no	3383 (97.0)	105 (3.0)	*P* = 0.193	3383 (95.7)	153 (4.3)	*P* = 0.047	3206 (88.4)	421 (11.6)	*P* = 0.005
	yes	1155 (97.7)	27 (2.3)		1155 (97.0)	36 (3.0)		1100 (91.3)	105 (8.7)	
Insomnia	no	2975 (96.7)	88 (3.3)	*P* = 0.018	2975 (96.4)	110 (3.6)	*P* = 0.408	2835 (89.3)	339 (10.7)	*P* = 0.966
	yes	828 (98.2)	40 (1.8)		828 (95.8)	36 (4.2)		782 (89.3)	94 (10.7)	
Chronic headache	no	3161 (96.9)	101 (3.1)	*P* = 0.388	3161 (94.3)	186 (5.7)	*P* = 0.000	3009 (89.6)	349 (10.4)	*P* = 0.065
	yes	986 (99.0)	26 (1.0)		986 (96.8)	33 (3.2)		931 (87.6)	132 (12.4)	

Table 4 showed result of logistic regression analysis of the association of various lifestyle factors with hyperthyroidism, hypothyroidism and positive TPOAb. We set whether or not having thyroid dysfunction as the dependent variable (yes = 1, no = 0) and lifestyle factors with significant value in Student’s t-test and chi-square test as independent variables for multiple unconditioned logistic regression analysis. For hyperthyroidism, alcohol consumption was found to be associated with decreased probability of disease incidence (OR [95% CI] =0.433 [0.219–0.857]), whereas increased daily cigarettes consumption was found to be associated with increased risk, though the trend was weak (OR [95% CI] = 1.045 [1.019–1.073]). For hypothyroidism, exercise (OR [95% CI] = 0.678 [0.467–0.984)]), alcohol consumption (OR [95% CI] =0.522 [0.351–0.775]) and having chronic headache (OR [95% CI] =0.665 [0.469–0.943]) were found to be associated with decreased probability of developing the disease, whereas BMI (OR [95% CI] =1.072 [1.020–1.126]) appeared to correlate positively with risk. When TPOAb positivity was analyzed, we found undergoing weight watch (OR [95% CI] = 0.730 [0.579–0.921]), smoker (OR [95% CI] =0.758 [0.595–0.966]), or alcohol consumption (OR [95% CI] = 0.754 [0.606–0.937]) was each associated with decreased risk whereas BMI (OR [95% CI] =0.661.030 [1.000–1.061]) was associated with increased risk. Interestingly, increasing daily staple amount appeared to correlate with decreased risk of positive TPOAb, although the effect is moderate (OR [95% CI] = 0.956 [0.918–0.995]). The collinearity of the variables in the regression equations were assessed with collinearity diagnostics. The characters of Tolerance, Eigenvalue and Condition Index indicated that the variables in the three regression equations did not have multicollinearity.

**Table 4:** Multivariate logistic regression analysis of risk factors of hyperthyroidism, hypothyroidism and positive of POAb

	Factor	OR	OR (95CI)	*P* value
Hyperthyroidism	alcohol consumption	0.433	(0.219,0.857)	0.016
	daily cigarettes	1.045	(1.019,1.073)	0.001
	insomnia	2.234	(0.689,7.247)	0.181
	education	1.247	(0.779,1.995)	0.357
	constant	0.022		< 0.001
Hypothyroidism	BMI	1.072	(1.020, 1.126)	0.006
	alcohol consumption	0.522	(0.351,0.775)	0.001
	exercise	0.678	(0.467,0.984)	0.041
	chronic daily headache	0.665	(0.469,0.943)	0.022
	smoker	0.702	(0.443,1.112)	0.131
	weight concern	0.770	(0.510,1.164)	0.215
	constant	0.026		< 0.001
TPOAb positive	daily staple	0.956	(0.918,0.995)	0.028
	BMI	1.030	(1.000,1.061)	0.047
	alcohol consumption	0.754	(0.606,0.937)	0.011
	smoker	0.758	(0.595,0.966)	0.025
	weight watch	0.730	(0.579,0.921)	0.008
	high school education	0.799	(0.627,1.020)	0.071
	on diet	0.784	(0.615,1.000)	0.050
	constant	0.147		< 0.001

## Discussion

### The epidemiological features of thyroid status in *She* ethnic minority

Among the 5154 subjects included in the research survey, the overall prevalence of thyroid dysfunction was 6.72% (with 2.72 and 4% for hyper- and hypo-thyroidism, respectively, as shown in the result section in Table 1). The TPOAb positive rate was 10.7% (Table 1). The trend was consistent with a recent large-scale epidemiological survey in China [14]. In male, there was no significant difference between the prevalence of hyperthyroidism and hypothyroidism. In female, the incidence of hypothyroidism and TPOAb positive were significantly higher than that of hyperthyroidism (Table 1). With the increase of age, the incidence of hyperthyroidism and hypothyroidism both increased in male, which was consistent with the survey in other countries [15].

### Alcohol consumption

The effect of alcohol consumption on AITD is elusive. Some previous research found no association between alcohol consumption and Graves’ Disease [16], while others suggested that moderate alcohol consumption might reduce the risk of Graves’ disease [17]. Autoimmune thyroid disease seems to be much more dependent on environmental factors than hitherto anticipated. A population-based case-control study in Denmark likewise observed that moderate alcohol consumption reduced the risk of overt autoimmune hypothyroidism [18]. In our study, alcohol consumption was found to be associated with reduced risk in hyperthyroidism, hypothyroidism and positive TPOAb (see Result section, Tables 3 and 4). The observed associations were independent of the type of alcohol (e.g. wine or beer), or gender. A protective effect of alcohol had also been reported for other autoimmune diseases such as rheumatoid arthritis, systemic lupus erythematosus and type 1 diabetes [19]. However, the mechanism of action of these protective effects of alcohol remains poorly understood.

### Smoking

The association between smoking and autoimmune thyroid disease (AITD) was explored by many epidemiological studies. It was confirmed that smoking is a risk factor for the development of Graves’ hyperthyroidism (GH), especially for Graves’ ophthalmopathy (GO) [20]. In consistence with these previous reports, our study also found smoking associated with increased incidence of hyperthyroidism. The association between smoking and hypothyroidism is less clear. According to a review by Vestergaard P. (2002), a number of previous studies failed to find any association between smoking and hypothyroidism [8]. However, several recent researches have provided strong evidence that smoking might reduce incidence of hypothyroidism [21, 22]. In our study, positive TPOAb was less prevalent in subjects who were smokers at the time of the study than it was in subjects who were not smoking (7.6% vs. 12.0%, *P* < 0.001). Similarly, hypothyroidism was less prevalent in smokers than it was in non-smokers (2.4% vs. 4.9%, *P* < 0.001). The mechanism of the protecting effect of smoking for hypothyroidism is unclear. Some research indicated that nicotine might play a role. Nicotine was thought to increase Tregs-mediated immune suppression of lymphocytes via α7 nicotinic acetylcholine receptor (α7 nAChR) [23]. Cigarettes smoking exposure was believed to inhibit Th1 cytokine production and may lead to enhancement of Th2 response [24]. Since Hashimoto’s thyroiditis was regarded as predominantly a disease caused by increased Th1 production, while Graves’s disease may be caused by the enhancement of Th2 response, the suppressive effect of cigarette smoking on Th1 response and the resulting enhancement of Th2 response may explain the decreased risk of hypothyroidism and increased risk of hyperthyroidism among smokers [25]. The effect of the smoking on the hyperthyroidism and hypothyroidism is interesting. Further research is required to better understand the underlying mechanisms.

### Exercise, weight watch and BMI

Little has been done to examine potential impact of exercise and physical activities on thyroid function. Studies in healthy, well-trained male athletes had shown that short-term high intensity exercise increased levels of circulating thyroid hormones [26]. In contrast, for long-term exercise, a study of men after six months of endurance training reported that free T4 concentrations were slightly decreased, with no change in TSH [27]. In our study, we did not find any correlation between exercise and hyperthyroidism. However, in the case of hypothyroidism, the prevalence was lower in the subjects who exercised compared to those who did not (3, 8% vs. 5.5%). In the logistic regression analysis, exercise was found to be associated with risk reduction in hypothyroidism, although no association in any direction was found between exercise and positive TPOAb. These results indicate that regular physical activity may be a protective factor for hypothyroidism. In our research we also found that subjects on weight watch had lower BMI as expected (22.686 ± 3.037 vs. 22.961 ± 3.463, *P* = 0.014), and lower prevalence of positive TOPAb and hypothyroidism (Table 3). However, there was no correlation between hyperthyroidism and weight watching or BMI. Many studies had reported that obesity can cause autoimmune disorders and increased TOPAb level, thus increases prevalence of hypothyroidism [28]. Our results were consistent with those reported in previous research.

### Daily staple, diet and salt intake

Globally, iodine supplementation has been the primary method to prevent mental retardation caused by iodine deficiency. However, in the recent few decades, there was a concern that excessive iodine exposure in some individuals may increase the incidence of autoimmune thyroiditis. Some began to question mandatory iodization of salt. In our research, we found no difference in the prevalence of hyperthyroidism, hypothyroidism or positive TPOAb among subjects’ groups with different amount of iodized salt consumption.

In this survey, we found an interesting relationship between subject’s daily staple amount and their probability of developing TPOAb positive. It seemed that increased daily staple was correlated with lower risk of TPOAb positive (see Results section, Tables 3 and 4). Though the trend was moderate. The OR was 0.956 with 95%CI of 0.918–0.995. It was unclear how daily staple affects thyroid disease development. However, several studies had indicated that starvation could cause hypothyroidism. It was shown that during long term energy withdrawal, thyroidal superoxide anion was able to lower the thyroid hormones thyroxine (T4) and triiodothyronine (T3) and cause a hypothyroid state in pigeons [29]. It was possible that decreased staple intake might raise TPOAb level, thus provoke the development of hypothyroidism.

### Stress

It is well known that Stress was a provoking factor in the pathogenesis of Graves’s disease. Stress may also precipitate patients who are predisposed to autoimmune thyroid dysfunction [30]. However, the relationship between stress and hypothyroidism remains obscure. Two previous studies conducted by Pan [31] and Rossana [32], respectively, did not find association between life events and the development of thyroid dysfunction. Many life style factors can evoke stress. Previous researches had detected insomnia [33] and chronic daily headache [34] as chronic stressors that can result in health problems. In our study, we evaluated potential association of these two stress factors with thyroid dysfunction. We found hyperthyroidism was less prevalent in people who suffered from insomnia than it was in people free of insomnia, although the prevalence for hypothyroidism was similar between the two groups. This was surprising as stress is known to trigger Graves Disease. However, it was possible that different types and degrees of stress affect thyroid function differently and insomnia-induced stress might be very different from those studied in previous research. For chronic daily headache, we found no association between headache and hyperthyroidism. However, prevalence of hypothyroidism was reduced in people who suffer from chronic daily headache (5.7% vs. 3.2%). Again, the difference in the type and degree of stress investigated might explain the different observations in our study versus those of others.

### Limitation of the study

The study had certain limitations, including its lack of ability to infer causality due to the cross-sectional nature of the research design, as well as the use of a less stringent *p* value to define statistical significance. Also, we used TSH level as the sole criteria for classifying thyroid dysfunction, which could potentially misclassify people with pituitary disease and resistance to thyroid hormone (though with very small probability) as having thyroid dysfunction. Further, the associations between lifestyle factors and blood TPOAb presentation were not analyzed under different thyroid disease status due to the design and scope of the study. Additional investigation would be needed to shed light on the complex interactions between lifestyle factors, TPOAb, and development of different thyroid diseases. Despite these limitations, the study provided useful epidemiological data for thyroid dysfunction research.

## Conclusion

Within the Chinese *She* ethnic minority, we found associations between different lifestyle factors and the incidence of different thyroid diseases. Although some of the factors in this study may be Chinese *She* ethnic minority specific, the research provided much more information about the life style factors and the thyroid dysfunction. Continued accumulation of such epidemiological data is necessary towards a better understanding and management of thyroid dysfunction.

## Data Availability

The datasets used in the analyses described in this study are available from the corresponding author on reasonable request.

## Abbreviations

AITD: Autoimmune thyroid diseases
BMI: Body mass index
DBP: Diastolic blood pressure
HDL: High-density lipoprotein-cholesterol
HLA: Human leukocyte antigen
HR: Heart rate
LDL-c: Low-density lipoprotein-cholesterol
SBP: Systolic blood pressure
T3: Triiodothyronine
T4: Thyroid hormones thyroxine
TC: Total cholesterol
TG: Triglycerides
TPOAb: Thyroid peroxidase antibody
TSH: Thyroid Stimulating Hormone
UA: Uric acid
